# Supplementary material for: Population-based cohort data used to assess trends in early resumption of sexual activity after voluntary medical male circumcision in Rakai, Uganda
Source: PLoS One. 2024 Nov 21;19(11):e0297240. doi: 10.1371/journal.pone.0297240 (PMC11581352; doi:10.1371/journal.pone.0297240)
Supplement: Attachment — Submitted filename: Response to Reviewers.pdf [file pone.0297240.s001.pdf]

Point by point

S/N	Comments	Solution
SPECIFIC COMMENTS:		
1	Reviewer #1: Title: The title is ok. However perhaps word "early resumption" rather than just resumption of sexual activity is what is being studied so this should be inserted. The study method needs to be added - a cross sectionals survey	The title has been re-written having both early and the methods
2	Abstract: The Abstract strikes me as been too lengthy and lacking in flow, there are too many brackets which interrupt the flow of the text, making the abstract difficult to read and assimilate easily. These should be minimized and only the essential information need be provided.	The abstract has been refined and some brackets have been removed to ease the flow and understanding
3	Introduction: This is mainly providing the justification for the study, without stating the background of the study population and the study objective. This should included some description of RCCS which is instead in the study methods. The study objective should be provided at the end of the introduction, this is missing.	The RCCS description has been added and the overall objective included. These portions were removed from the methods section.
4	Methods: The is some disparity between the WHO recommendation of 6 weeks to resume sexual activity and the MOH of Uganda's recommendation of 42 days. Some comments and justification should be provided on this. It is a little unclear how if the study is presumed to have been done in 2024 or 2023 how recent circumcision (<3 years) would have included those who had VMMC in cohorts (2013-2015, 2016 to 2018 etc). Unless these surveys were summated, this should be made clear. It is not also clear if these participants were surveyed at the study time or the information was just pooled from the previous surveys in data base, because there is an issue of memory or recall Biase then in these participants. The authors need to make this clearer.	Thank you so much for this important comment. Yes, there is a clear difference between WHO and Ugandan MOH definitions 6 weeks vs 42 days. However, for this particular study, our outcome was defined based on the Ugandan MOH definition of 42 days.<br>The recent circumcision (<3 years) was based on the particular year of the survey e.g survey one happened between 2013 and 2015 therefore the recent circumcision was <3 years on the day of interview or data collection for each survey visit. The most recent survey happened between 2018 and 2020. We have included this in our definition We understand and acknowledge this limitation (recall bias) and we have included this under study limitation.
5	Results: The descriptive data which provides general information of the study population is missing. The results go straight into the analytic data. The results are presented in an unyielding manner with long tables with statistical numbers which give the impression of raw data. The results should be presented better and some descriptive data should be provided.	Thank you so much for this observation. We have included the descriptive data for the study population.
6	Discussion: This discussion is good. However the explanation that the decline in ESR is based on improvement in counselling services is not supported by this study itself. The author ought to be able to show that this is the case in this study based on their	Thank you for this comment. We have adjusted this to fit our own data.

	own data. Some discussion on early resumption sex with condom use may be useful	
7	Conclusions<br>The conclusion is fair and accurate. However it adds little to what is already published, given the large cohort and the period studied the conclusion can be strengthen further.	Thanks, we have improved on the conclusion
**Reviewer 1**		
1	The title should be "Population-based Cohort…"	This has been changed to fit the suggestion
2	Line 7-8: A comma is missing: "Data from the Rakai Community Cohort Study, a cross-sectional study, were analyzed". Also you refer to men aged 15-49 years, but men are aged ≥18 years. I'd recommend you refer to these clients as "males".	Thanks a lot. This has been addressed
3	Line 9-10: You should delete the parent heses around the years of these studies.	Thanks, this has been removed
5	Line 13: For clarity with the 4 surveys, I would refer to this as "…participated in this analysis."	This has been corrected
6	Line 14: Previously you refer to 4 successive surveys, but here you refer to 3 surveys. Maybe say: "Across the first three surveys…" if that is what you mean here.	Thanks. This has been addressed
7	Line 20: The word "years" is missing after "45," and there is a word missing, as the rest of this sentence does not make sense.	Thank you so much. This has been corrected.
8	Line 21: Is the Adjusted Prevalence Ratio here 0? Also please check the CIs in lines 21-22, as something is wrong here.	Yes, the Prevalence Ratios are 0 and the CIs are also 0.0000018 and 0.0000026
9	Line 32: A comma is missing before "which".	Thank you , a comma has been added
10	Line 33: The word "for" is missing after "allow".	Thank you, the word for has been added
11	Line 42: These references are numbered out of order.	
12	Line 47: This is an 8-year period.	This was corrected as per tracked changes copy
13	General comment about the introduction section: There are several recent publications about sexual resumption following VMMC that have been excluded. I would recommend adding these. I also have a lot of remaining questions after reading the introduction like how many males have been circumcised for HIV prevention in Uganda? What is the specific guidance that circumcised males are given? Are there any particular challenges to Uganda that are related to wound healing or resumption of sexual activity?	The introduction section has been refined<br><br>Circumcised men in Uganda are advised to resume sex after 42 days or 6 weeks (each week having 7 days) as per ministry of health guidelines.
14	Line 51: A comma is missing before "which".	Corrected
15	Line 54-55 says that the men self-reported their circumcision status, but in line 62 refers to only medically circumcised men. It's unclear whether these males were identified through self-report or through medical records confirming their VMMC.	All the information about circumcision was self-report by the participants.<br>We have corrected this in the main document.
16	Line 85: Is the full name of this IRB the "Western IRB"?	Johns Hopkins University School of Medicine
17	Line 95: The word "years" is missing after "35-44".	The word years has been added
18	Line 99: "Fishermen" is one word.	Thanks, this has been addressed
19	Line 110: I'm not sure if this is a paragraph or a title.	This is a title and has been revised

20	Line 110-119: This doesn’t read as a cohesive paragraph in a Results Section.	This has been improved
21	Line 166: You should say “two-fold,” and this is an 8-year period.	This has been addressed
22	Line 179: Reference #19 is missing.	Thanks. The reference
23	Line 181-187: I would assume that more men aged 20-24 years are sexually active than males aged 15-19 years. Are there any data from Uganda that you could cite to support this?	Thanks, actually your assumption is correct and we have no data in Uganda that disagrees with that assumption. This has been corrected
24	General comment about the introduction and discussion section: More HIV-positive males that I would have expected were reported to have been circumcised and resumed sexual activity early. This is noteworthy as VMMC is usually promoted to males who are HIV-negative, though negative status is not required in order to be circumcised. The process for HTS before VMMC should be added to the introduction, and the fact that a high proportion of HIV-positive men were included in this analysis should be mentioned in the discussion section	The information has been added in the introduction
25	Line 193: If you think that VMMC status being only determined by self-report is a limitation, then that should be included in this paragraph.	Thanks, this has been addressed
26	Line 236: Reference #5 is incomplete.	This has been rectified. Thanks
27	This manuscript needs careful reviewing and proofreading throughout. Several sections lack specificity and detail, so more information is needed in the introduction and methods sections, especially. The results also needs careful attention.	Thanks
