# Supplementary material for: Population-based cohort data used to assess trends in early resumption of sexual activity after voluntary medical male circumcision in Rakai, Uganda
Source: PLoS One. 2024 Nov 21;19(11):e0297240. doi: 10.1371/journal.pone.0297240 (PMC11581352; doi:10.1371/journal.pone.0297240)
Supplement: Attachment — Submitted filename: Response to Reviewers.docx [file pone.0297240.s002.docx]

Point by point

| **S/N** | **Comments** | **Solution** |
| --- | --- | --- |
|  |  |  |
| **SPECIFIC COMMENTS:** | |  |
| 1 | Reviewer #1: The authors have adequately address all the key comments in the previous reviews in the areas of the abstract, the introduction, the methods, the results, the discussion and conclusions drawn | Thanks |
| 2 | Reviewer #2: 1. Typos in the Methods and Results sections of the Abstract | We have corrected typos in the methods and results sections |
| 3 | In line 21, it’s awkward that you refer to men with “no sexual relationship” reporting resuming sexual activities. I’d recommend you clarify the language to make it clear that you’re referring to men who were not married or otherwise in relationships. | We corrected this, the confusing statements removed. |
| 4 | In line 34, your references are incorrect. You are missing the Gray manuscript from 2007, the Auvert manuscript from 2004, and you should include the meta-analysis by Weiss from 2000. The current reference #2 looks to be incorrect as well. | We have corrected this and added these new references |
| 5 | In line 34, you must add that VMMC reduces the risk of heterosexual HIV acquisition by 50-60% in men. | This has been added |
| 6 | I’m not familiar with the recommendations by UNICEF, as mentioned in line 35. This should be cited or deleted. | This was sorted |
| 7 | It’s unclear if a new paragraph is supposed to start in line 56. | Yes this was a new paragraph |
| 8 | The introduction section is lacking some important information to orient the reader to VMMC and HIV prevention efforts in Uganda. The authors should include approximately how many males and the proportion of males that have been circumcised for HIV prevention in Uganda, what is the specific post-operative guidance that circumcised males are given regarding sexual activity post-VMMC (e.g., no vaginal sex, no sexual activity at all, no masturbation, etc.) Are there any particular challenges to Uganda that are related to wound healing or resumption of sexual activity that make adherence to the guidance particularly difficult? Note that this recommendation was included in the first review of this manuscript as comment #13. | This information has been included in the introduction |
| 9 | I assume that boys aged <18 years provided assent, not written consent, as noted in line 80. Please clarify the language here if the current language is not right. Same comment in line 100. | This has been clarified |
| 10 | **See the original comment #2.** Many study participants are aged <18 years, so referring to participants as “men” is incorrect. This issue has not been corrected throughout the manuscript. | This has been addressed |
| 11 | The paragraph from rows 104-120 contains many errors that makes it difficult to read and understand. The use of the terms “consistent” and “inconsistent” is awkward here. Perhaps readability would be improved if the authors used “similar” and “dissimilar”. | Addressed |
| 12 | The sentence in line 132 does not make sense. | Addressed |
| 13 | What is R19 in line 134? | Removed |
| 14 | Figure 1 (lines 175-176) has two titles. | Addressed |
| 15 | In Table 1, it appears as though survey round 4 (2018-2020) was significantly different than previous rounds (i.e., younger cohort, more post-primary education, differences in occupations, etc.). Also the largest decrease in ESR occurs in survey round 4. Is it possible that these cross-sectional cohorts include younger men that are different than prior surveys? The possible reasons for this should be discussed in the discussion section. | We have included a potential explanation in discussion section |
| 16 | What is the target that is mentioned in line 242? This should be included here. | Basically, WHO recommends zero persons resuming sex before the recommended period |
| 17 | The list of references look inconsistent with each other and with the journal’s instructions. | Corrected |
| 18 | In general, this manuscript still has several issues. There are many typos and awkward word choices that affect its readability. Careful review and copyediting is needed before resubmission. | Addressed |
